# Defect Reduction in HEMT Epilayers on SiC Meta-Substrates

**DOI:** 10.3390/nano16030158

**Published:** 2026-01-23

**Authors:** Vin-Cent Su, Ting-Yu Wei, Meng-Hsin Chen, Chien-Te Ku, Guan-Shian Liu

**Affiliations:** 1 Department of Electrical Engineering, National United University, Miaoli 36003, Taiwan; redant1234@gmail.com (T.-Y.W.); mhc@nuu.edu.tw (M.-H.C.); 2 Materials and Electro-Optics Research Division, Chung-Shan Institute of Science and Technology, Taoyuan 32546, Taiwan; gsliu@ncsist.org.tw (C.-T.K.); gsliu@hotmail.com (G.-S.L.)

**Keywords:** meta-structures, meta-substrates, silicon carbide (SiC), gallium nitride (GaN), nanostructures, high electron mobility transistor (HEMT)

## Abstract

Dislocation reduction in gallium nitride (GaN) epitaxial layers remains a critical challenge for high-performance GaN-based electronic devices. In this study, GaN epitaxial growth on newly-developed 4H-Silicon Carbide (SiC) meta-substrates was systematically investigated to elucidate the role of surface pattern geometry in modulating dislocation propagation. A series of truncated-hexagonal-pyramid meta-structures with a fixed array period and varying pattern ratios (R) were designed and fabricated to enable controlled tuning of the effective surface morphology. Atomic force microscopy confirmed comparable surface flatness for all samples after epitaxial growth. Cathodoluminescence analysis revealed a non-monotonic dependence of defect density on R, indicating the existence of an optimal pattern geometry. Among all configurations, the outstanding sample exhibited the lowest defect density, achieving a 54.96% reduction in threading dislocations (edge + mixed) compared with a planar reference. Cross-sectional transmission electron microscopy further confirmed a substantially reduced dislocation density and clear evidence of dislocation bending and termination near the meta-structured regions. These results demonstrate that geometry-engineered 4H-SiC meta-substrates provide an effective and scalable strategy for dislocation modulation in GaN epitaxy on SiC meta-substrates, offering a promising pathway toward advanced GaN power and RF devices.

## 1. Introduction

First-generation semiconductors, represented by silicon (Si) and germanium (Ge), possess relatively narrow bandgaps (≤1.12 eV), which intrinsically limit their breakdown electric field strength and high-temperature operation. Second-generation compound semiconductors, such as gallium arsenide (GaAs) and indium phosphide (InP), provide higher electron mobility and superior high-frequency performance; however, their moderate bandgaps still result in constrained breakdown voltage and thermal robustness. In contrast, third-generation wide-bandgap semiconductors, including silicon carbide (SiC) and gallium nitride (GaN), exhibit substantially larger bandgaps exceeding 3 eV, along with enhanced critical electric field strength and thermal conductivity [1,2,3]. These intrinsic material advantages enable reliable operation of electronic devices at elevated junction temperatures, higher breakdown voltages, and significantly increased power density. Beyond electronic devices, wide-bandgap semiconductors have also been extensively employed in a wide range of optoelectronic and photonic applications, including laser diodes [4,5], light-emitting diodes [6,7], and planar optical components [8,9,10,11].

As illustrated by the power–frequency operating map [12], SiC devices dominate the high-power and high-voltage regime owing to their superior thermal conductivity (~4.9 W cm^−1^ K^−1^) and wide bandgap (~3.2 eV), enabling reliable operation under high current density and harsh thermal conditions. In contrast, GaN devices exhibit outstanding high-frequency performance, attributed to the formation of a high-density two-dimensional electron gas (2DEG) at the AlGaN/GaN heterointerface, which supports high electron mobility and fast switching. The complementary strengths of these two materials motivate the GaN-on-SiC platform, which simultaneously integrates the high-frequency advantage of GaN with the excellent thermal management and power handling capability of SiC substrates. Consequently, this hybrid material system is particularly attractive for advanced electronic applications requiring high power density, high switching frequency, and robust thermal reliability.

SiC exists in several polytypes, among which 3C-SiC, 4H-SiC, and 6H-SiC are the most extensively studied for electronic applications [13]. As summarized in the literature [14], 4H-SiC possesses the widest bandgap (~3.26 eV) among the commonly used polytypes, together with a high critical electric field strength (~2.8 MV cm^−1^) and superior electron mobility along the c-axis (~1200 cm^2^ V^−1^ s^−1^). In addition, 4H-SiC exhibits the highest electron-saturated drift velocity, which is highly favorable for high-power and high-frequency device operation. As a result, 4H-SiC has emerged as the preferred substrate material for advanced power electronics and GaN-on-SiC heterostructure devices.

However, heteroepitaxial growth inevitably introduces mechanical stress and crystalline defects due to lattice and thermal expansion mismatches between the epitaxial layer and the substrate [15]. When the elastic strain energy exceeds a critical threshold, stress relaxation occurs through the formation of crystallographic dislocations. Such defects act as carrier scattering centers and leakage paths, thereby degrading carrier mobility, breakdown performance, and long-term device reliability. Consequently, mitigating stress-induced dislocation formation is essential for realizing high-performance GaN-on-SiC electronic devices.

To mitigate the high threading dislocation density (TDD) induced by heteroepitaxial stress, several effective dislocation reduction strategies have been extensively developed. One widely adopted approach is the introduction of a nucleation or buffer layer between the substrate and the epitaxial GaN film [16]. In addition to buffer-layer engineering, patterned substrate techniques have proven highly effective in further reducing dislocation propagation. By introducing micro- or nano-scale surface patterns, such as cylindrical holes or cone-shaped structures, dislocation bending, annihilation, and lateral overgrowth are promoted, leading to a substantial reduction in TDD [17,18,19]. Therefore, the extension of these concepts to patterned 4H-SiC substrates has attracted increasing attention, offering a promising pathway toward high-performance and high-reliability GaN-on-SiC power and RF devices. Nevertheless, conventional patterned substrates typically employ a single, uniform pattern geometry over the entire wafer, resulting in a statistically random distribution of defects and strain even though the overall dislocation density can be reduced. Such approaches therefore lack the capability to locally modulate defect density and stress distribution across the substrate. Moreover, without modifying the epitaxial growth conditions, these conventional designs provide limited flexibility in further reducing defect density within the epitaxial layer or enabling spatially controlled defect engineering.

In this work, we focus on the integration of GaN epitaxial layers on newly-developed 4H-SiC meta-substrates to simultaneously address dislocation density and thermal management challenges. While 4H-SiC provides excellent thermal conductivity and high breakdown capability, lattice and thermal mismatches, during GaN growth inevitably induce stress accumulation and threading dislocation formation. Introducing an engineered surface topology on the 4H-SiC meta-substrates enable effective strain redistribution during epitaxy, promoting dislocation bending, annihilation, and lateral overgrowth. Unlike conventional patterned substrates that employ a uniform pattern over the entire wafer, the proposed meta-substrate incorporates regionally defined surface patterns, allowing localized modulation of stress and defect behavior. This spatially engineered design represents a key innovation of the newly developed meta-substrate approach. As a result, the propagation of threading dislocations into the active GaN layer can be significantly suppressed without compromising the intrinsic thermal advantages of the SiC substrate. This work offers a scalable and substrate-compatible pathway for realizing high-quality GaN-on-SiC heterostructures, which are critically important for high-power, high-frequency, and high-reliability electronic devices.

## 2. Design, Fabrication, and Inspection of Patterned Substrates

**Figure 1:**
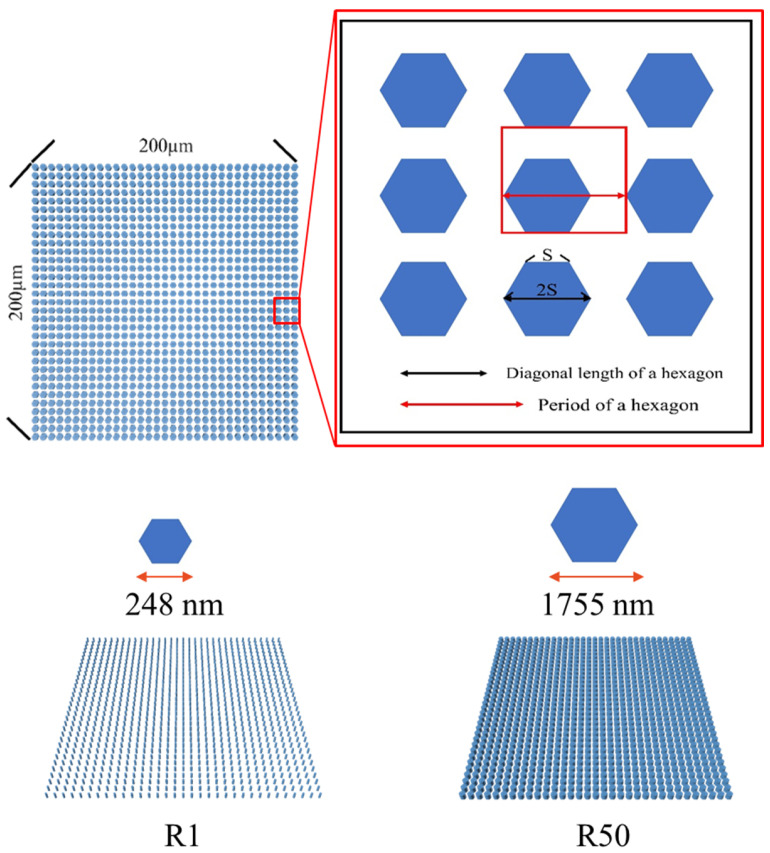
Design layout and geometric definition of the hexagonal-patterned structures on the 4H-SiC substrate. The left panel shows a representative large-area patterned region, while the right panel presents an enlarged schematic defining the geometric parameters of the hexagon.

To systematically investigate the influence of surface pattern geometry on epitaxial growth behavior and dislocation reduction, Figure 1 illustrates the design concept and geometric definition of the meta-structures on the 4H-SiC substrate. Each pattern configuration occupies an area of 200 μm × 200 μm, within which hexagonal features are periodically arranged. In total, eight distinct pattern configurations were implemented by systematically varying the relative feature size with respect to a fixed array period as shown in Table 1. Here, the parameter R denotes the ratio of the area occupied by a single truncated hexagonal feature (blue area) within one array period (red square), serving as a normalized descriptor of pattern density. For example, R1 corresponds to a ratio of 1%, while R50 represents a ratio of 50%. For each configuration, the corresponding diagonal length of the hexagon was determined based on the defined ratio and array period and is expressed as the diagonal length (2S) in nanometers, as summarized in Table 1. The left panel of Figure 1 shows a representative large-area patterned region, while the right panel presents an enlarged schematic defining the geometric parameters of the hexagon, including the side length (S), the diagonal length (2S), and the array period used in this study for the fabricated samples. The eight pattern-density values (R1–R8) were deliberately chosen to span a physically and technologically meaningful range of areal filling ratios, rather than to exhaustively sample all possible densities. From a fabrication perspective, increasing the pattern density beyond the highest R value investigated in this work leads to very narrow trench widths and reduced spacing between features, which imposes practical limitations on lithography fidelity, etch uniformity, and pattern transfer reliability, particularly over large areas. From a growth-physics standpoint, when the pattern density becomes sufficiently high, the surface morphology approaches that of a quasi-planar substrate, and the benefits of strain modulation and lateral overgrowth-assisted dislocation reduction are expected to gradually diminish or saturate. Each pattern regions are arranged in a regular array with a 1-mm separation between adjacent regions. All experimental measurements were performed at the center of each patterned region, where edge effects are minimized. The measurement area was selected according to the requirements of the specific characterization techniques. Because all pattern ratios are fabricated on the same wafer and processed under identical epitaxial growth conditions, the measured results directly reflect the influence of the pattern ratio itself rather than wafer-to-wafer or run-to-run variations.

It is worth noting that conventional patterned substrates typically employ a single, uniform pattern across the entire wafer, where one specific pattern geometry corresponds to one substrate design. In our work, multiple pattern ratios (R1–R50) are intentionally implemented as separate regions on the same SiC wafer, rather than on different wafers or samples. These patterned regions are separated by planar (unpatterned) buffer areas. Such a hybrid layout enables local modulation of strain and defect redistribution within the epitaxial layers, which is fundamentally different from a substrate uniformly patterned with a single geometry over the entire wafer. Moreover, the term “meta” refers to artificially designed and fabricated structures that do not occur naturally and whose properties arise from their engineered geometry rather than the bulk material itself. As a result, the proposed patterned substrate integrates multiple engineered pattern regions with distinct pattern ratios, functioning simultaneously to tailor epitaxial stress relaxation and defect behavior. Therefore, the term “meta-substrate” has been adapted to clearly distinguish this concept from conventional single-pattern SiC substrates.

**Figure 2:**
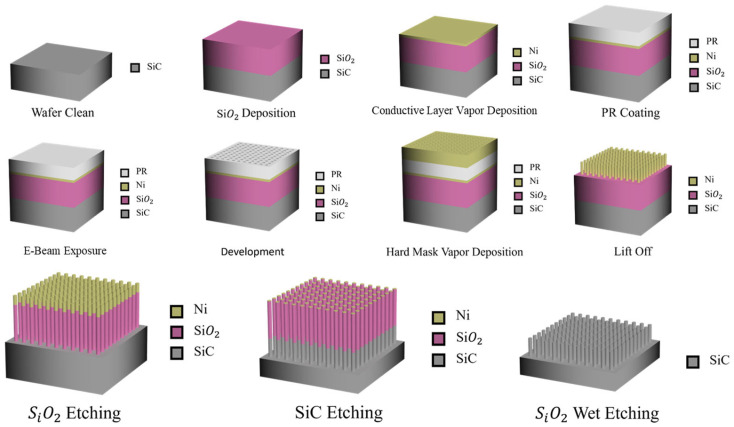
Schematic illustration of the fabrication process for truncated-hexagonal-pyramid-patterned 4H-SiC substrates.

The truncated-hexagonal-pyramid-patterned 4H-SiC substrates were fabricated through a sequence of standard micro-/nanofabrication processes, as schematically illustrated in Figure 2. The process began with a thorough cleaning of the 4H-SiC wafer, followed by the deposition of a silicon dioxide (SiO_2_) layer serving as an intermediate mask. A thin conductive layer was subsequently deposited to enable high-resolution electron-beam lithography, after which a photoresist layer was spin-coated onto the surface. The desired periodic pattern was then defined by electron-beam exposure and developed to form the resist template. A metallic hard mask was deposited, and a lift-off process was performed to transfer the pattern into the hard-mask layer. Using this patterned hard mask, the underlying SiO_2_ layer was selectively etched, followed by anisotropic dry etching of the 4H-SiC substrate to form truncated hexagonal pyramid structures with well-defined facets. Finally, the residual SiO_2_ mask was removed by wet etching, yielding a clean and precisely patterned 4H-SiC surface suitable for subsequent GaN epitaxial growth.

**Figure 3:**
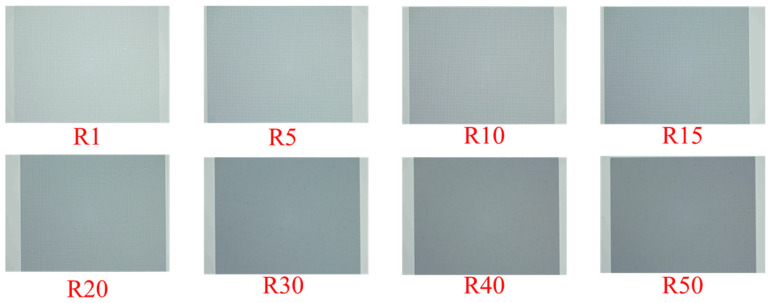
Optical microscopy (OM) images of the hexagonal-patterned 4H-SiC substrates with different pattern ratios (R).

Optical microscopy (OM) images of the hexagonal-patterned 4H-SiC substrates with different pattern ratios (R) are shown in Figure 3. All images were taken from representative areas within the 200 μm × 200 μm patterned regions. The samples correspond to R = 1, 5, 10, 15, 20, 30, 40, and 50, demonstrating uniform and well-defined pattern formation across the entire patterned area for all design configurations.

**Figure 4:**
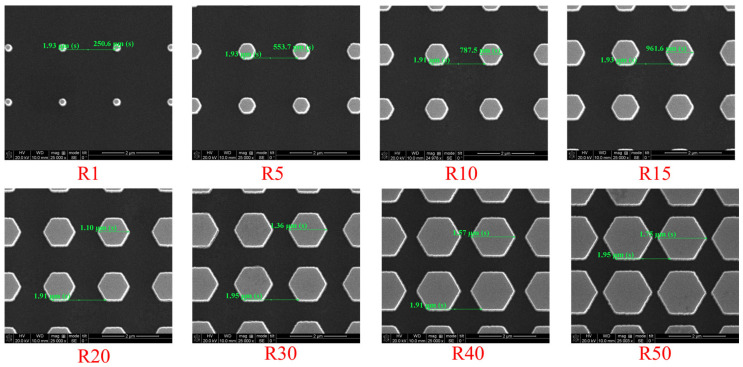
Top-view scanning electron microscopy (SEM) images of P2000 hexagonal-patterned 4H-SiC substrates with different pattern ratios (R = 1, 5, 10, 15, 20, 30, 40, and 50).

Top-view scanning electron microscopy (SEM) images of the P2000 hexagonal-patterned 4H-SiC substrates with different pattern ratios (R) are demonstrated in Figure 4. The samples correspond to R = 1, 5, 10, 15, 20, 30, 40, and 50, respectively. The images confirm well-defined hexagonal features with uniform shape and periodicity across all pattern configurations, as well as precise control of the feature dimensions in accordance with the designed ratios.

**Figure 5:**
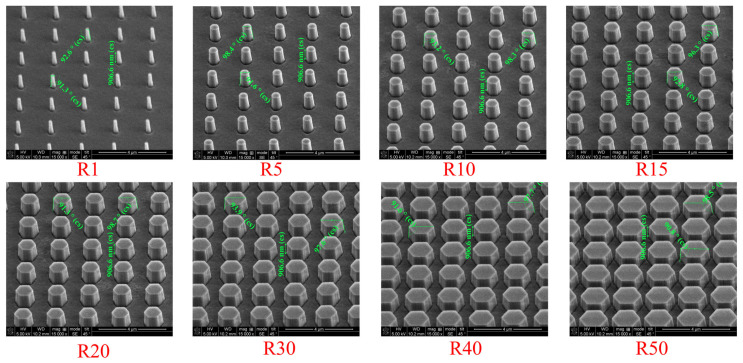
Tilted-view SEM images of P2000 hexagonal-patterned 4H-SiC substrates with different pattern ratios (R = 1, 5, 10, 15, 20, 30, 40, and 50).

Figure 5 shows tilted-view SEM images of the P2000 hexagonal-patterned 4H-SiC substrates with different R. As R increases from 1 to 50, the patterned features is densely packed truncated hexagonal pyramids, while preserving a highly ordered periodic arrangement. The tilted-view observation clearly reveals the three-dimensional morphology of the etched structures, including the truncated top surfaces and well-defined sidewall facets. Across all pattern configurations, the structures exhibit uniform height, consistent facet formation, and excellent pattern fidelity over the inspected regions. The measured lateral dimensions of the hexagonal features increase systematically with R, in good agreement with the designed geometric parameters summarized in Table 1. These results confirm precise control over both lateral geometry and three-dimensional morphology, providing a reliable template for subsequent GaN epitaxial growth on patterned 4H-SiC substrates. The height of the truncated hexagonal pyramids is approximately 900 nm for all R values, as confirmed by tilted-view SEM images in Figure 5.

## 3. Epitaxial Wafers and Characterization

After substrate patterning and surface preparation, the samples were subsequently subjected to epitaxial growth using metal–organic chemical vapor deposition (MOCVD). A GaN-based heterostructure was grown on the patterned 4H-SiC substrates. The epitaxial stack consists of a 50 nm AlN nucleation/buffer layer deposited directly on the patterned 4H-SiC substrate, followed by a thick GaN buffer layer with a thickness of approximately 2–2.1 μm. An ultrathin AlN interlayer (~1 nm) was inserted to improve interface quality, followed by a 25 nm AlGaN barrier layer and a 2 nm GaN cap layer. This layer design was employed to ensure high crystalline quality, effective strain management, and stable formation of a high-density 2DEG at the AlGaN/GaN interface. The same epitaxial structure and layer thicknesses were used for all patterned substrates to enable a fair comparison of geometry-dependent growth behavior. For the MOCVD growth parameters, trimethylgallium (TMGa), trimethylaluminum (TMAl), and ammonia (NH_3_) were used as the Ga, Al (and In) group-III and N group-V precursors. The AlN layer is grown at high temperatures of ~1050–1150 °C. Subsequently, the GaN buffer layer is grown at temperatures above ~1000 °C, with V/III molar ratios adjusted to balance adatom mobility, surface morphology, and defect density. For AlGaN barrier and GaN cap layers, similar conditions (elevated temperatures and V/III molar ratios optimized for composition and surface quality) are used, ensuring coherent interfaces and high crystal quality across all patterned regions.

The patterned samples and the planar reference were grown in adjacent consecutive MOCVD runs under identical growth conditions, using the same reactor, precursor chemistry, temperature, pressure, and gas flow settings. The planar reference growth was performed immediately following the growth of the patterned samples, without any changes to the growth recipe. By conducting the growths in consecutive runs with unchanged parameters, run-to-run variations were minimized.

**Figure 6:**
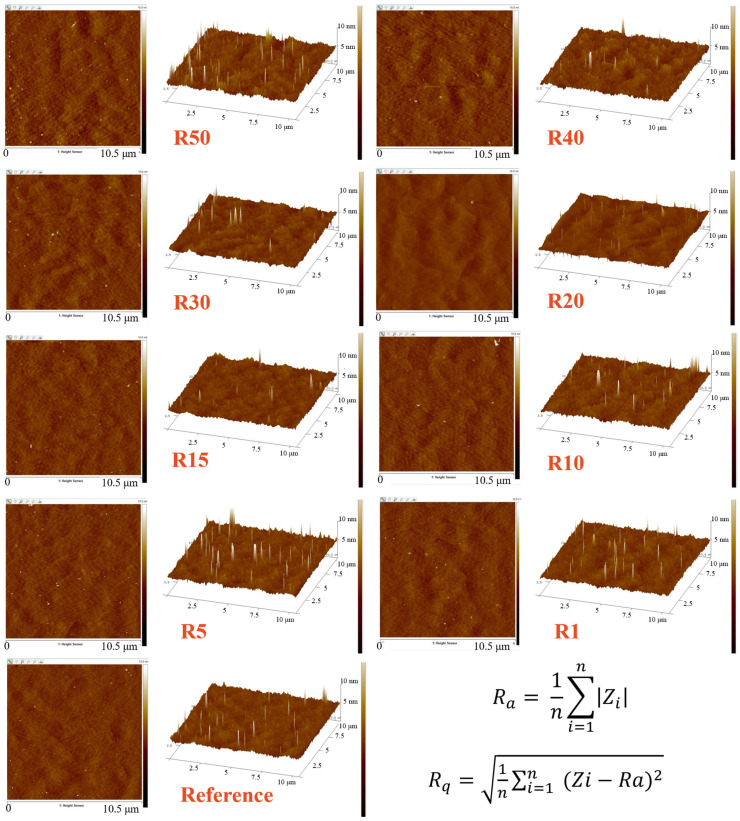
AFM-scanned images of P2000 hexagonal-patterned 4H-SiC substrates with different pattern ratios (R = 1, 5, 10, 15, 20, 30, 40, and 50).

After the completion of epitaxial growth, surface flatness was first evaluated to assess the morphological quality of the GaN epilayers, and atomic force microscopy (AFM) was therefore employed as the initial characterization technique. Figure 6 presents AFM measurements of the GaN epilayers grown on P2000 patterned 4H-SiC substrates with different R, together with a planar reference sample. Both two-dimensional height images and corresponding three-dimensional surface profiles were analyzed to evaluate the surface morphology after epitaxial growth. The arithmetic average roughness (R_a_) and root mean square roughness (R_s_) were extracted from the AFM data according to standard definitions. AFM measurements were performed at the center of each patterned region with a scan size of 10.5 µm × 10.5 µm. The RMS roughness values reported here were extracted from the corresponding AFM scans using identical analysis conditions. As summarized in Table 2, all samples exhibit sub-nanometer surface roughness, indicating high-quality GaN epitaxy on both patterned and reference substrates. A moderate dependence of surface roughness on the pattern ratio is observed, reflecting the influence of the underlying substrate topology on local growth kinetics and surface evolution. Importantly, no severe surface degradation is detected for any patterned configuration, confirming that the introduction of patterned 4H-SiC substrates does not compromise the surface smoothness of the overgrown GaN layers.

**Figure 7:**
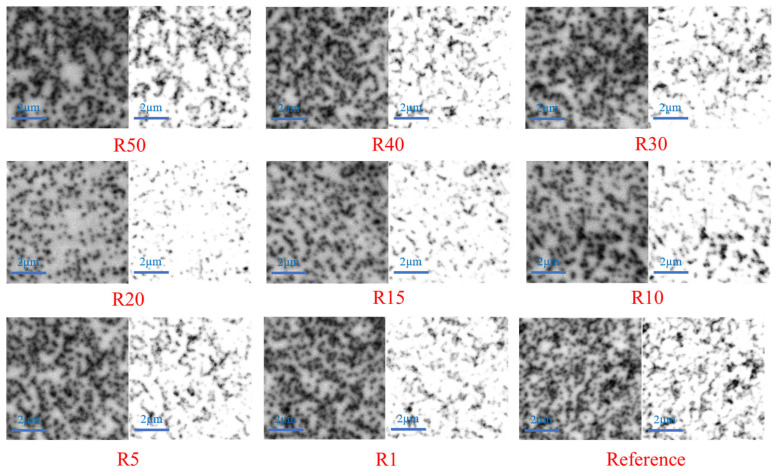
Plan-view cathodoluminescence (CL) images of GaN epilayers grown on P2000 patterned 4H-SiC substrates with different pattern ratios (R = 1, 5, 10, 15, 20, 30, 40, and 50), together with a planar reference sample.

To further evaluate the spatial distribution of crystalline defects following the epitaxial growth, cathodoluminescence (CL) measurements were conducted after confirming the surface flatness of the GaN epilayers by AFM. Figure 7 presents plan-view CL images of GaN epilayers grown on P2000 patterned 4H-SiC substrates with different pattern ratio (*R*), together with a planar reference sample. For each pattern configuration, the left image shows the original CL intensity map, whereas the right image shows the corresponding binarized black-and-white image, generated by applying an intensity threshold to distinguish defect-related non-radiative recombination centers from the background. The dark regions in the binarized images correspond to defect sites, enabling quantitative evaluation of defect density. Compared with the planar reference sample, the patterned substrates exhibit distinct variations in defect distribution and density as a function of R, indicating that the surface pattern geometry plays a critical role in modulating dislocation propagation during epitaxial growth. CL measurements were carried out under identical acquisition conditions for all samples. CL maps were recorded over a 7 µm × 7 µm scan area using an accelerating voltage of 30 kV at room temperature. The CL signal was collected with identical detector settings and acquisition time for all measurements.

**Figure 8:**
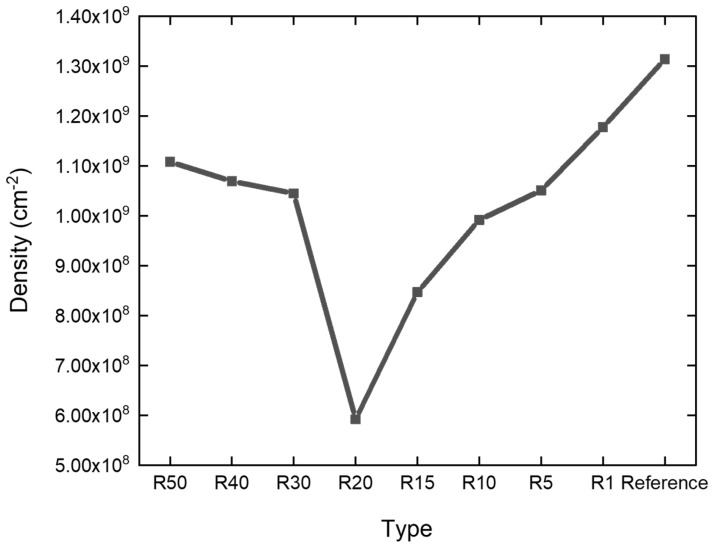
Defect density extracted from CL measurements for GaN epilayers grown on P2000 patterned 4H-SiC substrates with different pattern ratios (R), together with a planar reference sample.

To quantitatively evaluate the effect of surface pattern geometry on defect suppression, the defect density was extracted from the binarized CL images for all P2000 patterned samples and the planar reference. Figure 8 summarizes the CL-derived defect density as a function of the pattern ratio (R). A non-monotonic dependence of defect density on R is clearly observed. For very small R values, although the surface morphology geometrically approaches that of a planar substrate, the patterned regions still experience material removal due to etching, resulting in a reduced effective thickness compared with the reference sample. For the SiC meta-substrates fabricated using a positive electron-beam resist, the unmasked regions are etched, resulting in a reduced effective substrate thickness. At smaller R values, a larger etched fraction leads to greater effective thickness reduction, which influences strain relaxation and promotes higher dislocation density during GaN epitaxy. Consequently, the defect density for small-R patterns is only comparable to, but not identical with, that of the planar case. In contrast, at large R values, the patterned features become densely packed and behave effectively as a thick quasi-planar layer, leading again to defect densities close to the reference sample. Between these two limiting cases, an optimal pattern configuration emerges. Among all designs, the R20 sample exhibits the lowest defect density, reaching approximately $6.0\times 10^{8}\;{\mathrm{cm}}^{-2}$, which corresponds to a 54.96% reduction in threading dislocations (edge + mixed types) compared with the reference sample. This behavior indicates that an appropriately engineered pattern geometry can effectively modulate dislocation propagation by promoting dislocation bending and annihilation, whereas insufficient or excessive patterning diminishes this effect. The binarization threshold used for defect counting was determined based on the CL intensity histogram of the planar reference sample, where defect-related dark regions can be clearly distinguished from the surrounding area. Importantly, the same binarization threshold was applied to all samples, including all pattern ratios and the planar reference, to ensure a consistent and unbiased comparison of defect densities. In GaN-based heterostructures, dark spots observed in CL images are widely attributed to non-radiative recombination centers associated with threading dislocations (TDs), including edge, screw, and mixed dislocations. This correlation has been well established in the literature through combined CL, TEM, and selective etching studies, where dark CL contrast spatially coincides with dislocation cores [20,21]. We acknowledge that CL alone cannot unambiguously distinguish between different types of threading dislocations (edge, screw, or mixed), nor can it completely exclude contributions from point defects. As a result, in the present work, the dark features identified and counted in the CL maps are assumed to primarily correspond to threading dislocations.

## 4. Discussion

**Figure 9:**
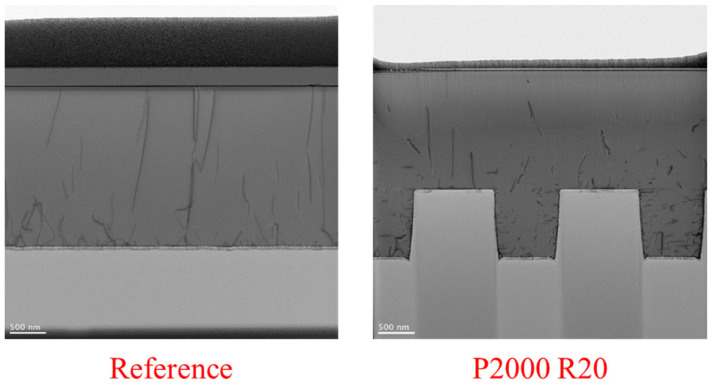
Cross-sectional transmission electron microscopy (TEM) images comparing the planar reference sample and the optimized P2000 R20 patterned 4H-SiC substrate.

To directly validate the defect reduction behavior inferred from CL measurements, cross-sectional transmission electron microscopy (TEM) was performed on the planar reference sample and the patterned substrate exhibiting the best defect suppression, namely the P2000 R20 configuration. Figure 9 compares representative TEM images acquired under identical imaging conditions. In the reference sample, a high density of threading dislocations propagating vertically from the GaN/SiC interface toward the surface is clearly observed. In contrast, the P2000 R20 sample exhibits a significantly reduced number of threading dislocations, with many dislocations showing termination or bending near the patterned regions. Based on direct dislocation counting within the TEM observation window, the threading dislocation density of the reference sample was estimated to be $1.96\times 10^{9}\;{\mathrm{cm}}^{-2}$, whereas that of the P2000 R20 sample was reduced to $2.79\times 10^{8}\;{\mathrm{cm}}^{-2}$. This corresponds to an approximately one-order-of-magnitude reduction in dislocation density, in excellent agreement with the trend obtained from CL-based defect density analysis. These results provide direct microstructural evidence that the optimized patterned substrate effectively suppresses dislocation propagation during GaN epitaxial growth. While the TEM results are consistent with the trend observed in the CL-based defect density analysis, additional TEM investigations on multiple samples would be required to rigorously confirm a one-order-of-magnitude reduction in dislocation density and to establish the generality of this behavior. It should be noted that, in the present work, TEM characterization was performed on representative samples only; therefore, the discussion is limited to the observed trends rather than definitive quantitative proof.

Moreover, detailed luminescence spectral analysis, which is highly effective for identifying defect-related emissions and evaluating material perfection [22,23], will be pursued in future studies to further substantiate the present spatially resolved CL observations. What is more, the discrepancy between the defect density for R20 obtained from CL and that derived from TEM is mainly attributed to the different inspection areas and sampling statistics of the two techniques. CL mapping probes a relatively large surface area, providing an area-averaged estimate of optically active non-radiative recombination centers. In contrast, TEM analysis is performed on a highly localized region, typically requiring sample dicing and thinning to a width below ~100 nm to directly observe dislocation distributions. As a result, the TEM-derived defect density may not fully capture large-area spatial variations. Despite this difference, it is important to note that the defect densities obtained from CL and TEM are within the same order of magnitude, indicating good overall consistency between the two characterization methods. In the present study, the discussion is limited to the structural and optical quality of GaN-based HEMT epilayers. Electrical characterizations, such as Hall-effect or transport measurements, will be pursued in future work to further evaluate the impact of defect reduction on device-relevant properties.

Based on the experimental results and established understanding of GaN epitaxy on patterned substrates, we attribute the observed reduction in defect density to a geometry-induced modulation of strain relaxation and growth dynamics. The geometry-engineered SiC meta-substrates introduce local variations in surface topography and effective substrate thickness, which modify the early-stage nucleation and subsequent GaN growth behavior. Because all samples share identical epitaxial structures and growth parameters, and the same trend is consistently observed across different pattern ratios, the effect is considered reproducible and primarily governed by geometric and strain-related factors intrinsic to the meta-substrate design. We emphasize that this interpretation is based on experimentally observed trends and widely accepted growth mechanisms, and that further studies involving additional structural characterization and modeling would be valuable to fully quantify and validate the proposed mechanism.

## 5. Conclusions

The growth of GaN-based HEMT-relevant layers on 4H-SiC meta-substrates was investigated to elucidate the role of geometry-engineered surfaces in dislocation modulation. By varying the pattern ratio (R) under a fixed array period within the meta-substrate architecture, a non-monotonic dependence of defect density on surface geometry was observed. At small R values, the locally patterned regions approached a quasi-planar morphology, where etching-induced thickness reduction limited the effectiveness of defect suppression. In contrast, at large R values, densely packed features behaved as thick quasi-planar layers, resulting in defect densities comparable to those of the planar reference. An optimal meta-substrate configuration was identified at R = 20, which achieved a 54.96% reduction in threading dislocations (edge + mixed), as revealed by cathodoluminescence analysis. Cross-sectional transmission electron microscopy further confirmed a substantially reduced dislocation density, accompanied by clear evidence of dislocation bending and termination near the patterned regions. These results demonstrate that meta-substrate–enabled, geometry-engineered patterning provides an effective and scalable strategy for controlling dislocation propagation in GaN epilayers on SiC substrates, offering a promising pathway toward high-performance GaN-based electronic devices.

## Figures and Tables

**Table 1:** Definition of the hexagonal pattern configurations used in this study.

P2000	R1	R5	R10	R15	R20	R30	R40	R50
Diagonal length of a hexagon (nm)	248	555	785	961	1110	1359	1570	1755

**Table 2:** Surface roughness parameters extracted from atomic force microscopy (AFM) measurements of GaN epilayers grown on P2000 patterned 4H-SiC substrates.

P2000	R1	R5	R10	R15	R20	R30	R40	R50	Ref
Ra (nm)	0.294	0.276	0.246	0.179	0.226	0.251	0.244	0.237	0.231
Rq (nm)	0.437	0.446	0.348	0.234	0.305	0.374	0.381	0.328	0.326

## Data Availability

The data regarding the findings of this study are available from the corresponding author upon reasonable request.
